# Spinal Lymphoma Presenting as an Epidural and Retropleural Mass With Concomitant Pathologic Compression Fracture: A Case Report

**DOI:** 10.7759/cureus.31155

**Published:** 2022-11-06

**Authors:** Nathan K Leclair, Hiromi Terai, Mitch Paro, Michael Blechner, Abner Gershon, Kevin Becker, Hilary Onyiuke

**Affiliations:** 1 School of Medicine, University of Connecticut School of Medicine, Farmington, USA; 2 Pathology and Laboratory Medicine, University of Connecticut Health, Farmington, USA; 3 Radiology, University of Connecticut Health, Farmington, USA; 4 Oncology, University of Connecticut Health, Farmington, USA; 5 Neurological Surgery, University of Connecticut Health, Farmington, USA

**Keywords:** spine neurosurgeon, neurology and neurosurgery, spine surgury, cns lymphoma, spine oncology

## Abstract

Lymphoma has traditionally earned the nickname “the great mimicker”. Its presentation as a primary spinal tumor is rare, and therefore seldom included in the differential diagnosis. However, its mimicking nature and diverse presentation make it very difficult to exclude entirely. Here, we present an elderly patient with histology-confirmed spinal lymphoma presenting as both an epidural mass with transforaminal extension into the retropleural space as well as vertebral body compression fracture, together leading to severe spinal stenosis and compressive myelopathy. Additional non-malignant compression fractures found in our patient allow for an interesting discussion on disease presentation and imaging-based diagnosis. We discuss our approach to diagnosis, surgical treatment, and post-operative medical care.

## Introduction

Primary spinal cord lymphomas are rare, accounting for only 1.7% of all primary spinal cord tumors in adults [1]. Furthermore, spinal epidural lymphomas account for a minority of all extranodal non-Hodgkin’s lymphomas (eNHL), with some reports estimating less than 1% [2,3] present as a primary spinal column tumors. Pathologically, spinal eNHL are frequently aggressive diffuse B-cell neoplasms that may have a rapidly deteriorating course. Spinal eNHL typically presents with back pain and symptoms of spinal cord compression including paresis/paraplegia, paraesthesia, and bowel/bladder incontinence [4-7]. However, they can rarely present with B-symptoms [4], or symptoms associated with compression of other structures [8].

In addition to spinal cord compression from epidural mass effect, spinal lymphomas can invade the vertebral column leading to pathologic fractures [9]. Furthermore, bony involvement in the spine can precipitate chronic symptoms following therapy [9]. Current treatment strategies for spinal lymphomas involve a multidisciplinary approach including neurosurgical intervention of epidural masses and/or stabilization of the spinal column, followed by medical therapy including the R-CHOP regimen (rituximab, cyclophosphamide, doxorubicin, vincristine, prednisolone), R-EPOCH regimen (rituximab, etoposide, oncovin, cyclophosphamide, doxorubicin), and radiotherapy [10,11].

Lymphoma is colloquially referred to as the “great imitator/mimicker” of central nervous system disease [12,13], given its low incidence and presentation resembling other primary or metastatic spinal tumors. Here, we describe an interesting case of primary spinal lymphoma presenting as an epidural mass with extension through the neural foramina and co-existing pathologic vertebral body compression fracture. This case highly illustrates the mimicking nature of spinal lymphoma and provides discussion for diagnostic and treatment paradigms.

## Case presentation

Clinical presentation

A 92-year-old female with no known significant past medical history presented to the hospital with progressive mid-back pain and weakness that started six months prior and has progressed in severity. At the time of the presentation, she was unable to ambulate independently. She did not report any bladder or bowel incontinence, or saddle anesthesia. Neurologic exam revealed decreased strength in bilateral lower extremities (3/5 on left, 2/5 on right). Workup at the time included an abdominal CT that demonstrated a chronic compression fracture of L4.

Neuroradiology

**Figure 1:**
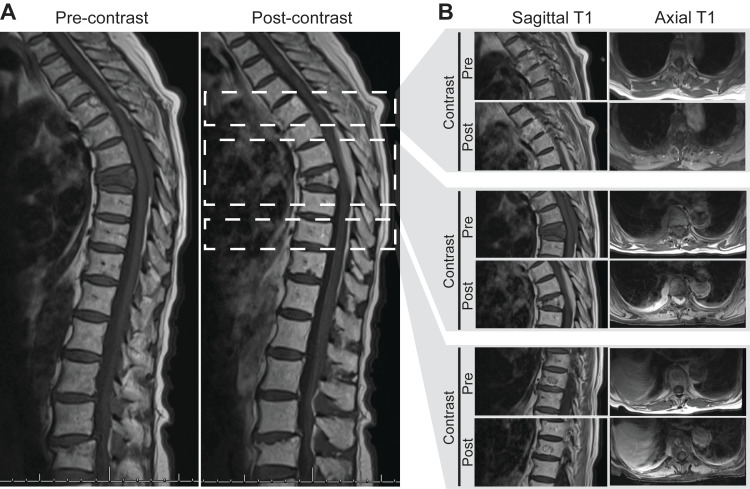
Spinal axis characterization of multiple vertebral and epidural lesions. A) Midline sagittal cuts of pre- and post-contrast T1-MRIs at the time of patient presentation. B) Representative sagittal and axial T1-MRIs pre- and post-administration of contrast agent. Slices are taken at levels indicated in (A).

**Figure 2:**
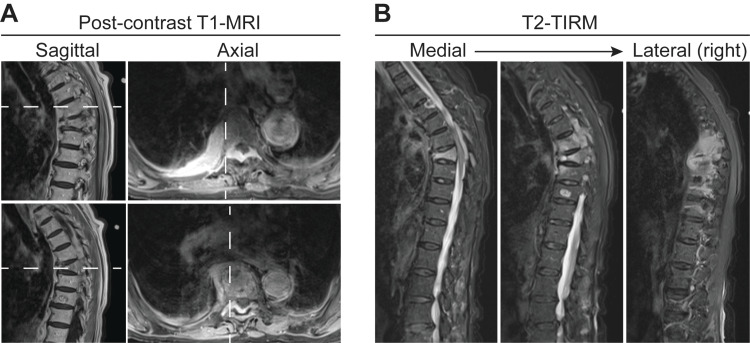
MRI characterization of primary spinal lymphoma with epidural, retropleural, and vertebral body involvement. A) Sagittal and axial post-contrast T1-MRIs demonstrating extensive homogenously enhancing soft tissue mass in the epidural space extending retropleurally through the right neural foramina at multiple levels from T7 – T9. Dashed lines represent relative location of sagittal and axial cuts. B) Fat saturated T2 turbo inversion recovery MRI (TIRM) cuts from medial to right lateral demonstrating hyperintense signal in the T7 – T9 vertebral bodies, indicative of lymphoma extension into the bone. Epidural and retropleural lymphoma appears hypointense to cerebrospinal fluid.

MRI of the thoracic spine revealed a severe compression fracture of the T8 vertebral body with abnormal hypointense signal on T1-weighted images and abnormal enhancement following administration of gadolinium-based contrast agent (GBCA) (Figure 1). Additionally, there was a homogenously enhancing soft-tissue mass in the posterior epidural space at the level of the T6 through T9 vertebrae which, through the neural foramina, was contiguous with a right paraspinal mass (Figure 2A). The compression fracture and adjacent soft tissue mass were hyperintense compared to normal bone and muscle on fat-saturated, T2-weighted, inversion recovery MRI (TIRM) (Figure 2B). The spinal cord was compressed between the retropulsed posterior wall of the T8 vertebral body and the posterior epidural mass. A preliminary diagnosis of metastatic cancer to the spine was made. Additionally, there were enhancing lesions noted at T1 and T10, believed to be benign vertebral hemangiomas. There was also a compression fracture of the L4 vertebra which however demonstrated no abnormal signal characteristics and no abnormal enhancement.

Surgical approach and tumor pathology

**Figure 3:**
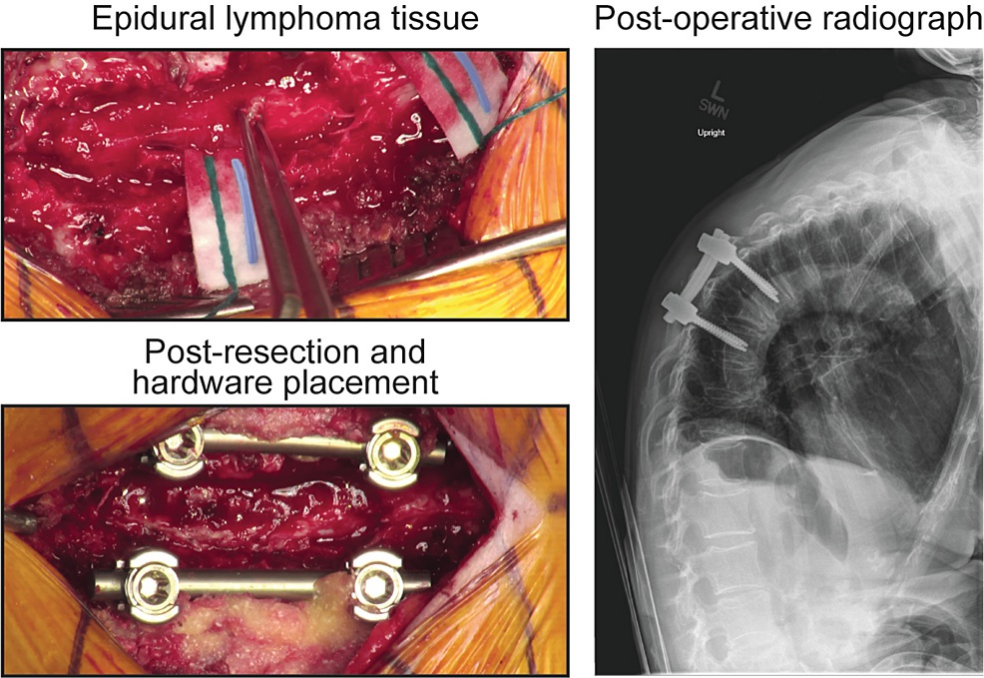
Intraoperative view of epidural lymphoma resection and decompressive laminectomy. T7 – T9 decompressive laminectomy was performed, and epidural lymphoma was observed and resected off the underlying dura. Instrumented fusion was performed to stabilize the spinal column. Post-operative radiograph confirmed stable hardware fixation of T7 – T9.

**Figure 4:**
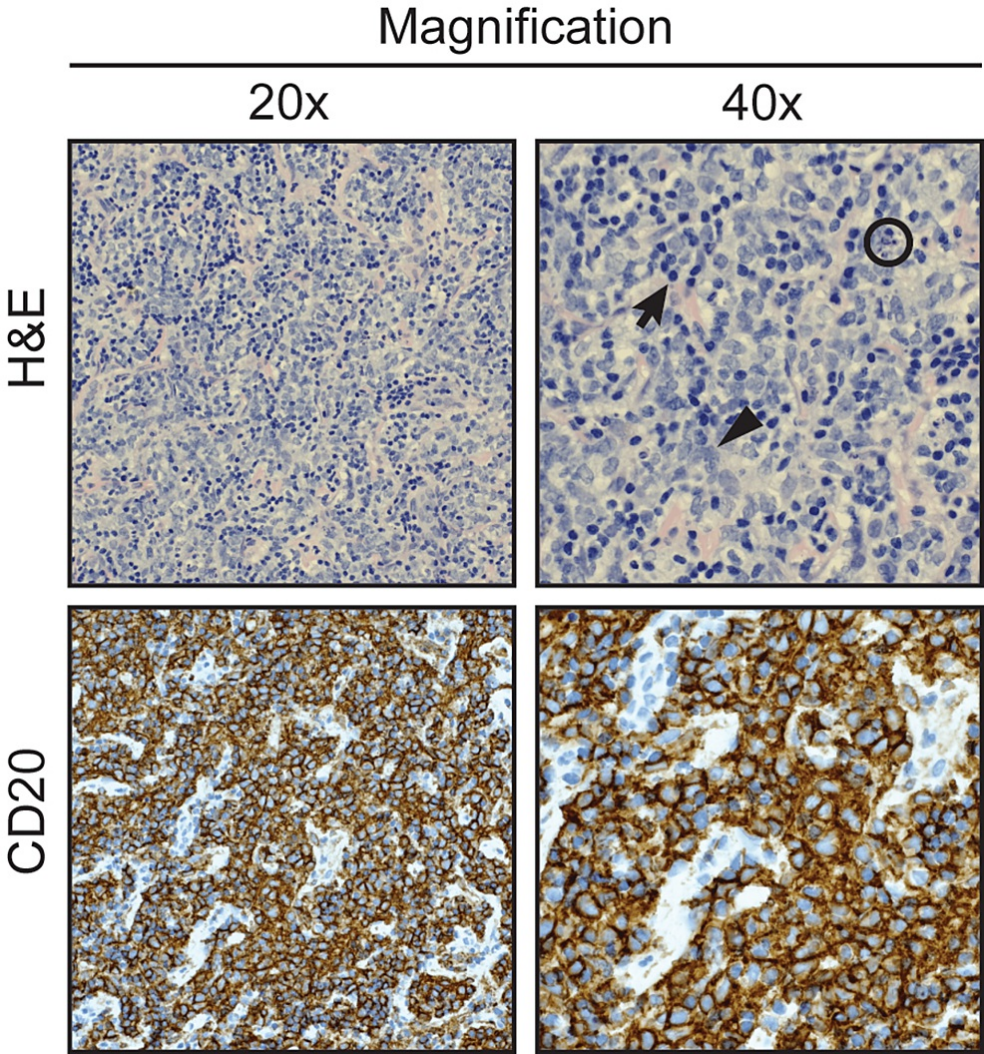
Histopathology of resected epidural spinal lymphoma. Histologic examination reveals an infiltrate of medium-to-large pleomorphic B-lymphocytes (large arrow). These cells exhibit irregularly clefted nuclei with open, dispersed chromatin. There are also numerous benign appearing, small T-cells admixed (small arrow). Numerous apoptotic cells are seen (circle). An immunohistochemical stain for CD20 confirms the B-cell lineage and accentuates the serpiginous pattern. Flow cytometric immunophenotyping confirmed a clonal population of B-cells with kappa light chain restriction. The final diagnosis was diffuse large B-cell lymphoma (DLBCL).

To alleviate spinal cord compression a decompressive laminectomy and instrumented fusion were performed at T7 - T9. The epidural mass was visualized and resected off the dura (Figure 3). Histologically, the extradural mass revealed a high-grade diffuse large B-cell lymphoma (DLBCL) with focal areas of fibrosis and numerous apoptotic and mitotic figures (Figure 4A). Neoplastic cells were immunoreactive for CD20, CD10, and BCL6, and demonstrated 50% MIB1 proliferation index. They were negative for BCL2, CD3, CD5, CD43, and cyclin D1 (Figure 4B).

Post-operative course

**Figure 5:**
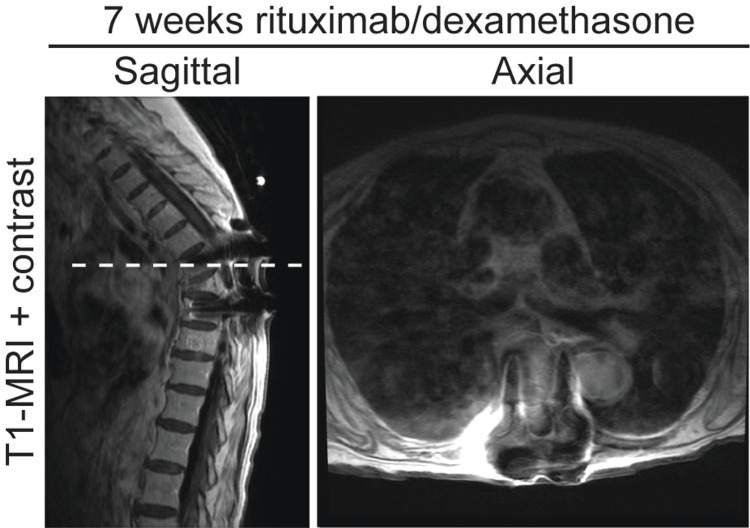
Tumor response to medical therapy with dexamethasone and rituximab. Contrast-enhanced T1-MRI sequences obtained seven weeks after initiating rituximab/dexamethasone medical therapy demonstrating tumor response to medical therapy. Dashed line represents level of axial cut. Study quality is limited by hardware artifacts.

Post-operatively the patient was started on moderate dose dexamethasone. She was offered treatment with conventional chemotherapy regimens including R-CHOP and R-EPOCH. However, these were not advised given the possibility of significant side effects in her advanced age. She elected for treatment with monotherapy rituximab, an anti-CD20 antibody that directly targets the B-cell lymphoma with minimal side effects. Rituximab (375mg/m^2^) was started with eight infusions at two-week intervals, followed by monthly maintenance infusions. An MRI obtained seven weeks after initiating rituximab therapy demonstrated positive disease response with resolution of the epidural mass and a decrease in size of the retropleural extension (Figure 5). Clinically, two months after surgery her back pain had improved but she remained wheelchair bound with weakness in the lower extremities improving with physical therapy.

## Discussion

We present a case of a high-grade DLBCL presenting as a pathologic thoracic vertebral body compression fracture and epidural mass with transforaminal extension - a rare presentation of the vertebral body, epidural, and retropleural involvement. While a few similar cases have been reported in the literature, this case report serves to expand upon best practices for diagnostic workup and management of primary spinal epidural lymphoma (PSEL) and further illustrate the importance of a wide differential diagnosis given the mimicking nature of spinal lymphomas specifically and lymphomas in general.

Vertebral body compression fractures are not uncommon in the elderly population [14]. Most often these are secondary to osteoporosis but may also be due to metastatic disease, multiple myeloma, or lymphoma. Complete replacement of the normal marrow of the vertebral body by a hypointense signal on T1-weighted images is more frequent in the presence of malignancy. Enhancement following GBCA tends to be more heterogenous and more intense in malignant fractures compared to osteoporotic fractures [15,16]. Given the fatty composition of bone marrow, fat-saturated MRI sequences can improve the detection of abnormal enhancement compared to non-fat-saturated sequences [15,16]. Our patient demonstrated the imaging characteristics of both types of compression fracture: her L4 compression fracture, likely from osteoporosis, did not enhance with contrast, while the tumor-infiltrating T8 compression fracture that was diffusely hypointense on T1-weighted MRI and demonstrated intense contrast enhancement (as seen in Figure 1) as well as hyperintensity on fat-saturated sequences (Figure 2B).

Further complicating the diagnosis, in this case, was the observed transforaminal extension of the tumor into the paraspinal space. This phenotype mimics that of peripheral nerve sheath tumors, schwannomas, ependymomas, or more rarely meningiomas, which can invade through neural foramina and present with symptoms of spinal nerve radiculopathy [17]. Schwannomas can show similar paraspinal and epidural space involvement [18], but are frequently benign in nature and do not induce malignant compression fractures.

The differential diagnosis of epidural masses is broad, including infectious processes, epidural hematoma, and primary malignancies. Aside from imaging studies, pathologic evaluations and clinical findings are crucial in differentiating these etiologies. Epidural masses may go unnoticed until they cause symptomatic cord compression. The cord compression seen in this patient was likely multifactorial and due to the large compressive epidural tumor (lymphoma), thoracic spine instability, and exaggerated thoracic kyphosis secondary to the T8 compression fracture. Clinical judgment to operate was guided by the established Spinal Instability Neoplastic Score (SINS) [18,19], characterizing this patient’s lesion as potentially unstable. In addition to predicted instability, the patient’s neurological deficits, mainly bilateral lower extremity weakness, prompted surgical intervention including resection of the epidural mass and spinal stabilization. Whilst this may have been a somewhat risky operation given this patient’s age, prompt surgical stabilization led to the arrest of further neurological decline in addition to reduced pain level, and finally, does represent a good surgical indication [20]. Surgical resection also provided tissue for final diagnosis and treatment options. Previous studies found no difference in overall survival between medical and surgical management of spinal lymphomas, and indeed better outcomes in functional status with medical management alone [11]. However, with objective signs of spinal cord compression, many advocate for surgical resection and decompression to improve functional status [21,22]. Additionally, R-CHOP regimens carry high toxicity in elderly patients and require dose adjustments that compromise efficacy [23,24]. Hence, our approach here was to stabilize the spine, debulk the tumor, and treat post-operatively with dexamethasone/rituximab alone to avoid additional harm from standard chemotherapy regimens.

## Conclusions

Spinal lymphomas remain a diagnostic challenge to the clinician, in part due to their rarity and propensity to mimic other pathologies. MRI, especially pre-contrast T1-weighted and post-contrast fat-saturated sequences, can help differentiate malignancy-associated pathological compression fractures from osteoporotic compression fractures and is especially important in elderly patients where these are frequent findings. The decision to operate on elderly patients with spinal lymphomas should be a multidisciplinary one. The surgeon must weigh the need for rapid spinal cord decompression versus the morbidity of surgery. These tumors can respond well to rituximab, which should be considered as monotherapy in patients unable to tolerate conventional chemotherapy.
